# Supplementary material for: The effects of ischemia during rest intervals on strength endurance performance
Source: PLoS One. 2023 Apr 6;18(4):e0280231. doi: 10.1371/journal.pone.0280231 (PMC10079082; doi:10.1371/journal.pone.0280231)
Supplement: Attachment — Submitted filename: Responses to Review.docx [file pone.0280231.s002.docx]

PONE-D-21-36446

The effects of ischemia during rest intervals on strength endurance performance

Reviewer #1: This is an interesting study where authors investigate the effects of ischemia applied before the first set and between sets of resistance exercise (bench press) performed to failure on number of repetitions, time under tension and bar velocity. The authors conclude that ischemia applied between-sets does not increase strength-endurance nor bar velocity during bench press exercise performed to muscle failure.

I hope that my comments be helpful to improve the readability of the manuscript.

**Reply - We are grateful for the commitment and very valuable comments that helped us to improve the quality of the manuscript. We hope that the revised manuscript adequately addresses all the raised issues.**

General comments

The manuscript is well written and easy to follow. Although I am not a native English speaker, I suggest some minor adjustments in writing. The objective is clear and the results are important to the practitioner. Some caution must be exercised when trying to speculate the results from acute study to long term adaptations. I believe the discussion section needs some work to improve the interpretation of the results.

Specific comments.

Introduction

I would not say that ischemia is the same as blood flow restriction, as ischemia may result from the external compression, but it is not the method.

**Reply – yes we agree with the reviewer that ischemia may result from external compression, that's why we made such a change to: “The ischemia-induced by the external compression or by the BFR cuffs”**

L 64-65 - What do the authors mean by “The differences between those methods are related to the point when ischemia is applied”. This sentence refers to using restriction continuous, intermittent or pre-conditioning.

Also, when BFR is used during resistance training, the aim is to partially blood inflow and fully restrict blood outflow in the exercising muscles. This is not the aim of the ischemic pre-conditioning. So I suggest that this paragraph be rewritten.

**Reply - I have corrected the indicated sentence**

L62- replace combine with combined

**Reply – the change has been made**

L67 – I suggest that “ischemic pre-conditioning” be used instead of “ischemia pre-conditioning”

**Reply – the change has been mad**

L68 – please use the same term – ischemia intra-conditioning (or ischemic, if you choose to replace), but do not use “intra-conditioning ischemia”. The same comment applies to other places in the manuscript where “intra-conditioning ischemia” is used.

**Reply - Thank you, I have standardized the term throughout**

L75 – I suggest that “… it can be assumed…” be replaced with “… it is possible that…”.

**Reply – the change has been made**

L77 – performed

**Reply – the change has been made**

L79 – ischemic pre-conditioning. I suggest that the authors include when the ischemic pre-conditioning was applied. It is possible that some readers are not familiar with this strategy.

**Reply – we added such information about when the ischemia was applied**

L83 -replace “a” with “the”

**Reply – the change has been made**

L86 -replace “a” with “the”

**Reply – the change has been made**

L89 – Please include what was assessed as physical performance?

**Reply – the change has been made**

L91-93 – In this study, ischemia was applied not only between sets, but also previous to the beginning of the training sets.

**Reply - I added information on application of ischemia also before the first series**

L94-95 – Please rewrite the sentence starting with “It was hypothesized…”

**Reply – the sentence was rewritten**

Methods

This section is easy to follow.

I wonder why bar velocity and time under tension were assessed if the objective was to investigate the effects of ischemic intra-conditioning in strength endurance performance. It is not in the introduction; they are not markers of strength endurance and is briefly discussed in the discussion section.

**Reply – time under tension is increasingly used as an indicator of exercise volume, more reliable than the number of repetitions, and the value of the maximal exercise volume performed is an important aspect in assessing strength-endurance performance. Further also changes in bar velocity may indicate the level of increasing fatigue. Therefore, both parameters can be important parameters in the assessment of the impact of BFR on the level of strength-endurance performance.**

L 158- coefficient

**Reply - word was corrected**

L182-184 – please, rewrite. It is confusing. What tests were used?

**Reply – sentence was corrected**

Results

Table 1 – specify what is present in parentheses. I believe it is the CI. Specify that the ES presented is Cohen’s d.

**Reply – yes it is CI. The table was corrected**

Discussion

For me, this is the critical part of this manuscript, and authors need to rewrite some of the information to improve flow and readability. For example, in the 2nd paragraph, authors mention the results of bar velocity and power and contrast them to the findings of strength endurance. These variables are very different.

Also, I suggest that authors organize the discussion according to the variables they investigate, and then try to make a final statement gathering all the discussion presented.

**Reply – The main problem with preparing the discussion is that there are no other studies about intra-conditioning and strength-endurance performance to which we could compare our results, so we used the results from other ischemia methods (ischemic pre-conditioning) or other measuring and other variables. However, as suggested by the reviewer, we have made changes to the discussion structure**

L228 - replace author’s with authors’

**Reply – done**

L 239 – 241 – There is a comparison with the study by Wilk who observed that ischemic intra-conditioning improved bar velocity is a training session consisting of 5 sets with 3 reps at 60% 1RM (maximal number of repetitions was not assessed). Why authors try to discuss the different set durations if the first study did not assess strength endurance?

**Reply - we decided to make this comparison because there are no other studies that have used ischemia only during rest intervals**

The important question that has to discussed is why was not there difference between experimental and control condition in the present study?

When authors bring the study of Wilk I imagined they would discuss the lack of effects in bar velocity. This is an interesting discussion. The highest peak velocity and mean velocity should have presented the same pattern in this study and Wilk’s, why it did not happen?

Time under tension decreased and number of repetitions was not affected, how do you explain that?

**Reply - as suggested by the reviewer, we have added appropriate explanations**

L47-L251 – This is important information and should be discussed further

**Reply - the discussion in this regard has been widened**

L252- I am not sure that physiological responses should be discussed here, as you did not assess them. But Why would physiological responses increase during ischemia condition?

Reply - yes, the reviewer is right, so we have removed the term potential increases of physiological responses during ischemia condition.

L257 – it is not just intra-conditioning that can have affected the results, but also the pre-conditioning.

**Reply – yes, we made appropriate changes**

L277 – replace do with does

**Reply – done**

L280- Please discuss the pressure applied

**Reply - Ghosh et al. does not provide such information therefore we decide to delete this ref. In other refs. we added relevant information**

L285 – delete was

**Reply – done**

L286- delete the

**Reply – done**

L310 – Present the limitation in a different paragraph.

**Reply – done**

L324 – 327 – This is speculation and the authors have no results to support it. It should not be in the conclusion.

**Reply - I agree with the reviewer's opinion, therefore I deleted the indicated sentence**

References

Please correct the references as there are no journal names.

**Reply - In fact, I haven't noticed such a big mistake. Thank you for paying attention**

6. PLOS authors have the option to publish the peer review history of their article (what does this mean?). If published, this will include your full peer review and any attached files.

If you choose “no”, your identity will remain anonymous but your review may still be made public.

Do you want your identity to be public for this peer review? For information about this choice, including consent withdrawal, please see our Privacy Policy.

Reviewer #1: Yes: Renato Barroso

[NOTE: If reviewer comments were submitted as an attachment file, they will be attached to this email and accessible via the submission site. Please log into your account, locate the manuscript record, and check for the action link "View Attachments". If this link does not appear, there are no attachment files.]

While revising your submission, please upload your figure files to the Preflight Analysis and Conversion Engine (PACE) digital diagnostic tool, https://pacev2.apexcovantage.com/. PACE helps ensure that figures meet PLOS requirements. To use PACE, you must first register as a user. Registration is free. Then, login and navigate to the UPLOAD tab, where you will find detailed instructions on how to use the tool. If you encounter any issues or have any questions when using PACE, please email PLOS at figures@plos.org. Please note that Supporting Information files do not need this step.

In compliance with data protection regulations, you may request that we remove your personal registration details at any time. (Remove my information/details). Please contact the publication office if you have any questions.
